# Prediction of neuroblastoma cell response to treatment with natural or synthetic retinoids using selected protein biomarkers

**DOI:** 10.1371/journal.pone.0218269

**Published:** 2019-06-12

**Authors:** Viera Dobrotkova, Petr Chlapek, Marta Jezova, Katerina Adamkova, Pavel Mazanek, Jaroslav Sterba, Renata Veselska

**Affiliations:** 1 Laboratory of Tumor Biology, Department of Experimental Biology, Faculty of Science, Masaryk University, Kotlarska, Czech Republic; 2 International Clinical Research Center, St. Anne’s University Hospital, Pekarska, Czech Republic; 3 Department of Pathology, University Hospital Brno and Faculty of Medicine, Masaryk University, Jihlavska, Czech Republic; 4 Department of Pediatric Oncology, University Hospital Brno and Faculty of Medicine, Masaryk University, Cernopolni, Czech Republic; University of South Alabama Mitchell Cancer Institute, UNITED STATES

## Abstract

Although the administration of retinoids represents an important part of treatment for children suffering from high-risk neuroblastomas, approximately 50% of these patients do not respond to this therapy or develop resistance to retinoids during treatment. Our study focused on the comparative analysis of the expression of five genes and corresponding proteins (DDX39A, HMGA1, HMGA2, HOXC9 and PBX1) that have recently been discussed as possible predictive biomarkers of clinical response to retinoid differentiation therapy. Expression of these five candidate biomarkers was evaluated at both the mRNA and protein level in the same subset of 8 neuroblastoma cell lines after treatment with natural or synthetic retinoids. We found that the cell lines that were HMGA2-positive and/or HOXC9-negative have a reduced sensitivity to retinoids. Furthermore, the experiments revealed that the retinoid-sensitive cell lines showed a uniform pattern of change after treatment with both natural and sensitive retinoids: increased DDX39A and decreased PBX1 protein levels. Our results showed that in NBL cells, these putative protein biomarkers are associated with sensitivity or resistance to retinoids, and their endogenous or induced expression can distinguish between these two phenotypes.

## Introduction

Neuroblastoma (NBL) is the most common extracranial solid tumor in children, accounting for 8-10% of childhood cancers in the USA and Europe [1], with an incidence of 10.5 per million children and affecting males slightly more often than females (1.2:1.0) [2]. NBL is typically heterogeneous, with variations in clinical appearance from spontaneous regression to aggressive metastatic disease disseminating mainly to the liver, bones, brain, and skin [3]. Children suffering from high-risk NBL have a very poor prognosis, with overall survival rates of only 40% [4], even though an aggressive multimodal therapy comprising induction chemotherapy, surgery, radiotherapy, high-dose chemotherapy with autologous stem cell transplantation, biologics and immunotherapy is administered to these patients.

In the last decade, differentiation therapy with retinoids in patients with minimal residual disease was proven to be effective, since such a treatment was reported to prevent tumor relapse after myeloablative therapy. Although retinoids as differentiation inducers represent an important part of high-risk NBL treatment, their toxicity and acquired resistance to retinoids limit their use in clinical practice [5]. Therefore, identifying molecular markers that can predict the therapeutic response to retinoids is an important aspect for their use in clinical practice [6].

Retinoids, which are natural and synthetic vitamin A derivatives, play a key role in the regulation of many processes in organisms, including cell differentiation, growth or apoptosis, which predetermines them for use in both cancer therapy and chemoprevention. All-*trans* retinoic acid (ATRA) and 13-*cis* retinoic acid (13-*cis*-RA) are the most frequently used retinoids in pediatric oncology, especially in the treatment of acute promyelocytic leukemia and high-risk NBL [7,8]. Intrinsic and acquired resistance to these compounds is a multifactorial phenomenon with the involvement of different mechanisms in the final resistance phenotype, making the proper identification and prediction of the cause of resistance in each individual even more challenging [9]. As the biological activity of retinoids depends on their binding to specific nuclear receptors (Retinoic acid receptor (RAR), Retinoid X receptor (RXR)), various alterations in their proper expression and function have been discussed as “upstream” markers indicating low or no response to retinoid treatment [10,11].

Although the administration of retinoids represents an important part of treatment for children suffering from high-risk NBL, approximately 50% of these patients do not respond to this therapy or develop resistance to retinoids during treatment [5]. The present study focused on the comparative analysis of the expression of genes and corresponding proteins (DDX39A, HMGA1, HMGA2, HOXC9 and PBX1) that have recently been discussed as possible predictive biomarkers of the clinical response to retinoid differentiation therapy [6].

DDX39A (DExD-Box helicase 39A) is an ATP-dependent RNA helicase that is involved in pre-mRNA splicing and is required for the export of mRNA out of the nucleus [12]. Another 2 candidate biomarkers are members of the high mobility group A (HMGA) protein family, which functions as ancillary transcription factors with a key role in controlling cell growth and differentiation: HMGA1 and HMGA2 [13]. In 2011, another putative biomarker of the response to retinoids was suggested by Mao and coworkers – HOXC9 (Homeobox protein Hox-C9), a member of the family of homeobox transcription factors that regulate morphogenesis and cell fate specification [14]. PBX1 (Pre-B-cell leukemia homeobox-1) is a member of another homeodomain protein family involved in a variety of processes, including cell differentiation and tumorigenesis [15]. PBX1 represents one of the key cofactors modulating the affinity and stability of HOX/DNA binding by heterodimerization with HOX proteins and has been suggested to induce neuronal differentiation [16,17].

To the best of our knowledge, this study was the first project based on multiple analyses of all these candidate biomarkers in one experimental study. Such an approach allowed us to judge the usefulness of these putative biomarkers together in the same subset of NBL cell lines and by one research group under the same experimental setting. Moreover, besides natural retinoids, including ATRA and 13-*cis*-RA, the most frequently administered retinoids in pediatric oncology, synthetic retinoids, another promising group of compounds for anticancer therapy, were also used in our project. In this study, we revealed that endogenous expression of some of the suggested biomarkers, especially when analyzed together, could predict the reduced ability of these cells to undergo retinoid-mediated differentiation.

## Materials and methods

### Tumor samples and derivation of cell lines

Tumor samples taken from 20 patients suffering from NBL (12 males, 8 females; age range at the time of diagnosis: 1 months – 19 years old) were included in this study. Written informed consent was obtained from each patient or patient’s parents. The Research Ethics Committees of the School of Medicine and the School of Science, Masaryk University (Brno, Czech Republic) approved the study protocol. Twenty NBL cell lines derived from these tumor samples were established in our laboratory with a previously described procedure [18].

### Immunohistochemistry

Formalin-fixed, paraffin-embedded (FFPE) surgical samples of neoplastic tissues were retrieved from the files of the Department of Pathology, University Hospital Brno, Czech Republic. The histologic sections stained with hematoxylin-eosin were reviewed by the same pathologist (MJ), and representative tissue blocks were selected for IHC analysis. 4-μm-thick sections from the blocks were cut, deparaffinized with pure xylene for 3× 5 min, washed in 96% alcohol for 3× 5 min and finally rinsed with distilled water. Endogenous peroxidase was inactivated by 3% H_2_O_2_ in methanol for 10 min, and the samples were then rinsed with distilled water; antigen retrieval was performed (incubation in citrate buffer of pH 6.0 at 98°C for 20 min followed by cooling for 20 min and washing in PBS for 3× 5 min), followed by incubation with primary antibodies in a wet chamber at room temperature for 1 h. Subsequently, the samples were rinsed with PBS for 3× 5 min. The EnVision+ System was used in accordance with the manufacturer's instructions in the wet chamber at room temperature for 45 min, followed by rinsing with PBS and visualization using 3,3'diaminobenzidine. Nuclei were counterstained using Mayer's hematoxylin for 1 min and bluing in water for 2-3 min for optimal results. Following dehydration in an ethanol series of increasing concentrations and clearing in xylene, the preparations were mounted onto Pertex. Positive and negative controls were evaluated in each IHC experiment. Negative controls consisted of slides processed without the primary antibodies. All of the antibodies used in this protocol and tissues that served as positive controls are described in the Table 1.

**Table 1:** Primary and secondary antibodies. Type: Mono, monoclonal; Poly, polyclonal. Host: Rb, rabbit; Mo, mouse; Ho, horse; Go, goat. Dilution (for methods): WB, Western blotting; IHC, immunohistochemistry. Localization of the antigen in positive controls: N, nuclear; C, cytoplasmic.

**Primary antibodies**						
Antigen	Manufacturer	Type / Host	Catalog No.	Dilution WB	Dilution IHC	Positive control IHC
DDX39A	Abcam	Mono / Rb	ab180857	1:1000	1:150	Human testis (N)
HMGA1	Cell Signaling Technology	Mono / Rb	12094S	1:500	1:500	Human colorectal cancer (N)
HMGA2	Cell Signaling Technology	Mono / Rb	8179S	1:500	1:200	Human colorectal cancer (N)
HOXC9	Abcam	Mono / Rb	ab50839	1:250	-	-
	Bioss	Poly / Rb	bs-7982R	-	1:100	Human kidney (N, C)
PBX1	LifeSpan Biosciences	Mono / Mo	LS-C133363	1:1000	1:50	Human pancreas (N)
GAPDH	Cell Signaling Technology	Mono / Rb	2118S	1:20,000	-	-
**Secondary antibodies**						
Specificity	Manufacturer	Host	Catalog No.	Dilution WB	Conjugate	
Anti-Mo IgG	Cell Signaling Technology	Ho	7076	1:5000	Horseradish peroxidase	
Anti-Rb IgG	Cell Signaling Technology	Go	7074	1:5000	Horseradish peroxidase	

For each of the evaluated proteins, specific nuclear or cytoplasmic immunostaining was considered positive. The percentage of antigen-positive tumor cells (TC) was counted and categorized into five levels: - (0% positive TC), +/- (1-10% positive TC), + (11-50% positive TC), ++ (51–80% positive TC), and +++ (81–100% positive TC). The intensity of immunostaining (immunoreactivity, IR) was classified as none (0), weak (1), medium (2), or strong (3). IR was also evaluated in cells of tissues, which were chosen as positive controls. The slides were evaluated with Olympus BX50 light microscope at ×200 magnification. At least 5 discrete foci of tumor tissue were analyzed, and the average staining intensity and the percentage of antigen-positive cells of the entire covered area were determined.

### Cell cultures

Twenty NBL cell lines derived in our laboratory from NBL tumor samples as described above and two reference NBL cell lines were included in this study. Both reference cell lines were purchased from the European Collection of Cell Cultures (ECACC): SH-SY5Y (ECACC cat. no. 94030304) and SK-N-BE(2) (ECACC cat. no. 95011815; MYCN amp.) and were reported as RA-sensitive [19]. The cells were grown in a 1:1 mixture of Dulbecco´s modified Eagle´s medium (DMEM) and Ham´s F 12 medium supplemented with 20% fetal calf serum, 2 mM glutamine, 100 IU/mL penicillin and 100 μg/mL streptomycin (all purchased from GE Healthcare Europe GmbH, Freiburg, Germany) and 1% nonessential amino acids (purchased from Biosera, Nuaille, France). The cell lines were maintained under standard conditions at 37°C in a humidified atmosphere containing 5% CO2 and subcultured 1-2 times weekly.

### Chemicals

ATRA and 13-*cis*-RA were prepared as stock solutions at a concentration of 100 mM in dimethyl sulfoxide (DMSO; Sigma); 9-*cis* retinoic acid (9-*cis*-RA), retinoic acid p-hydroxyanilide (Fenretinide, 4-HPR) and Bexarotene (BEX) were prepared at a concentration of 10 mM in DMSO (all purchased from Sigma Aldrich, St. Louis, MO, USA). Reagents were stored at -20°C under light-free conditions.

### Retinoid treatment

Stock solutions of all natural (ATRA, 9-*cis*-RA, 13-*cis*-RA) and synthetic (BEX, 4-HPR) retinoids were diluted in fresh cell culture medium to obtain final concentrations of 1 μM. Cells were seeded onto Petri dishes 24 h prior to treatment, and untreated cells were used as a control. Cell populations were individually treated with all the retinoids listed above and were harvested after seven days of cultivation.

### Cell proliferation

The proliferation activity of cell populations, both untreated and treated with retinoids was evaluated by MTT assay. For this purpose, cells were seeded in 96-well microtiter plates at a density of 2500 cells per well in the culture medium volume of 200 μl per well. After 24 hours, the fresh medium containing one of the five retinoids at the concentration of 1μM or a control medium was added. After seven days of cultivation, the culture medium was removed, and 200 μl of DMEM/Ham´s F-12 medium mixture (1:1) containing 3-[4,5-dimethylthiazol-2-yl]-2,5-diphenyltetrazolium bromide (MTT) (Sigma) at a final concentration of 455 μg/ml in medium was added to each well. The medium with MTT was replaced by 200 μl of DMSO per well after a 4-h incubation under standard conditions in order to solubilize the MTT product. Subsequently, the absorbance was measured at 570 nm with a reference absorbance at 620 nm wavelength using a Sunrise Absorbance Reader (Tecan). Obtained data were analyzed using one-sample T-test (two-tailed); p<0.05 (*) and p<0.01 (**) were considered statistically significant.

### RT-PCR

The relative expression levels of genes of interest were studied using RT-PCR, and total RNA for this purpose was extracted using the GenElute™ Mammalian Total RNA Miniprep kit (Sigma-Aldrich). RNA concentration and purity were determined spectrophotometrically, and equal amounts of RNA were reverse transcribed into cDNA using M-MLV reverse transcriptase (Top-Bio, Prague, Czech Republic). RT-PCR was carried out in 20-μL reaction volumes using the GeneQ thermal cycler (BIOER). To ensure that the amplification does not reach a saturation plateau, the suitable number of cycles for analysis of each gene in question was selected on the basis of comparison of RT-PCR products after 25, 30, 35 and 40 cycles of reaction (S1 Fig). According to this initial analysis, expression level of each gene was semi-quantified after the 30 cycles of reaction, i.e. in the exponential phase of amplification. The products were subsequently separated with 1% agarose gel electrophoresis and visualized in a UV transilluminator (UVITEC Cambridge). Visualized transcript bands were quantified using ImageJ. The reference genes *HSP90AB1* and *GAPDH* were used as an endogenous control. Data were analyzed using one-sample T-test (two-tailed); p< 0.05 was considered statistically significant. The primers used in this study are listed in Table 2.

**Table 2:** F, forward primer; R, reverse primer. Primers.

Gene symbol	Gene name	Primer sequence
*DDX39A*	DExD-box helicase 39A	F: 5´- GAACGGGGAGCCAGCATCAT - 3´ R: 5´-TTCTTAGGGGGAGCTGGTGT - 3´
*HMGA1*	High mobility group AT-hook 1	F: 5´-TCACTCTTCCACCTGCTCCT - 3´ R: 5´- TTGTTTTTGCTTCCCTTTGG - 3´
*HMGA2*	High mobility group AT-hook 2	F: 5´- GCAAGGCAACATTGACCTGAG - 3´ R: 5´- GCAAGGCAACATTGACCTGAG - 3´
*HOXC9*	Homeobox C9	F: 5´- AGCAAGCACAAAGAGGAGAAGG - 3´ R: 5´- TTCCAGCGTCTGGTACTTGGT - 3´
*PBX1*	PBX homeobox 1	F: 5´- CTCGGCTGGTGGATACCCTT - 3´ R: 5´- TGCGATTGCTGGGAGATCAG - 3´
*HSP90AB1*	Heat shock protein 90 alpha family class B member 1	F: 5´- CGCATGAAGGAGACACAGAA - 3´ R: 5´- TCCCATCAAATTCCTTGAGC - 3´
*GAPDH*	Glyceraldehyde-3-phosphate dehydrogenase	F: 5´- AGCCACATCGCTCAGACACC - 3´ R: 5´- GTACTCAGCGCCAGCATCG - 3´

### Western blotting and immunodetection

Whole cell extracts from all NBL cell lines were loaded onto 10% and 12% polyacrylamide gels and blotted on polyvinylidene difluoride membranes (Bio Rad Laboratories, Munich, Germany). The membranes were then blocked with 5% nonfat dry milk dissolved in phosphate buffered saline (PBS) containing 0.1% Tween-20 and incubated overnight with the corresponding primary antibody. After a 1-h incubation with a secondary antibody, proteins were visualized with the ECL-Plus detection system (GE Healthcare, Little Chalfont, UK). For all experiments, rabbit monoclonal anti-GAPDH served as a loading control. Data were analyzed using one-sample T-test (two-tailed); p< 0.05 was considered statistically significant. Both primary and secondary antibodies used in this study are listed in Table 1.

## Results

### Endogenous expression patterns of putative biomarkers were different in tumor samples and corresponding cell lines

**Fig 1:**
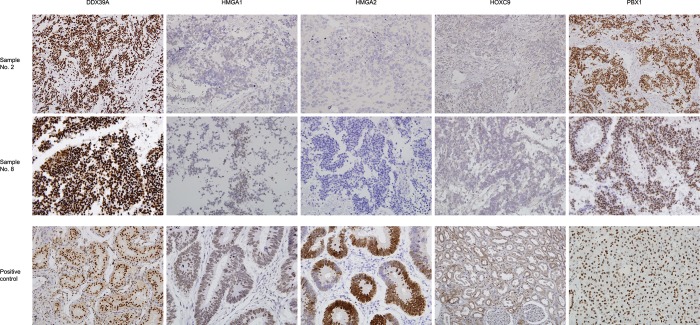
Representative IHC analysis of putative biomarkers in tumor samples. Representative expressions of DDX39A, HMGA1, HMGA2, HOXC9, and PBX1 in the tumor samples No. 2 (corresponding to the NBL-13 retinoid-sensitive cell line) and No. 18 (corresponding to the NBL-36 retinoid-resistant cell line). Representative expressions of these markers in positive control samples are also given for comparison. Original magnification, 200×.

**Fig 2:**
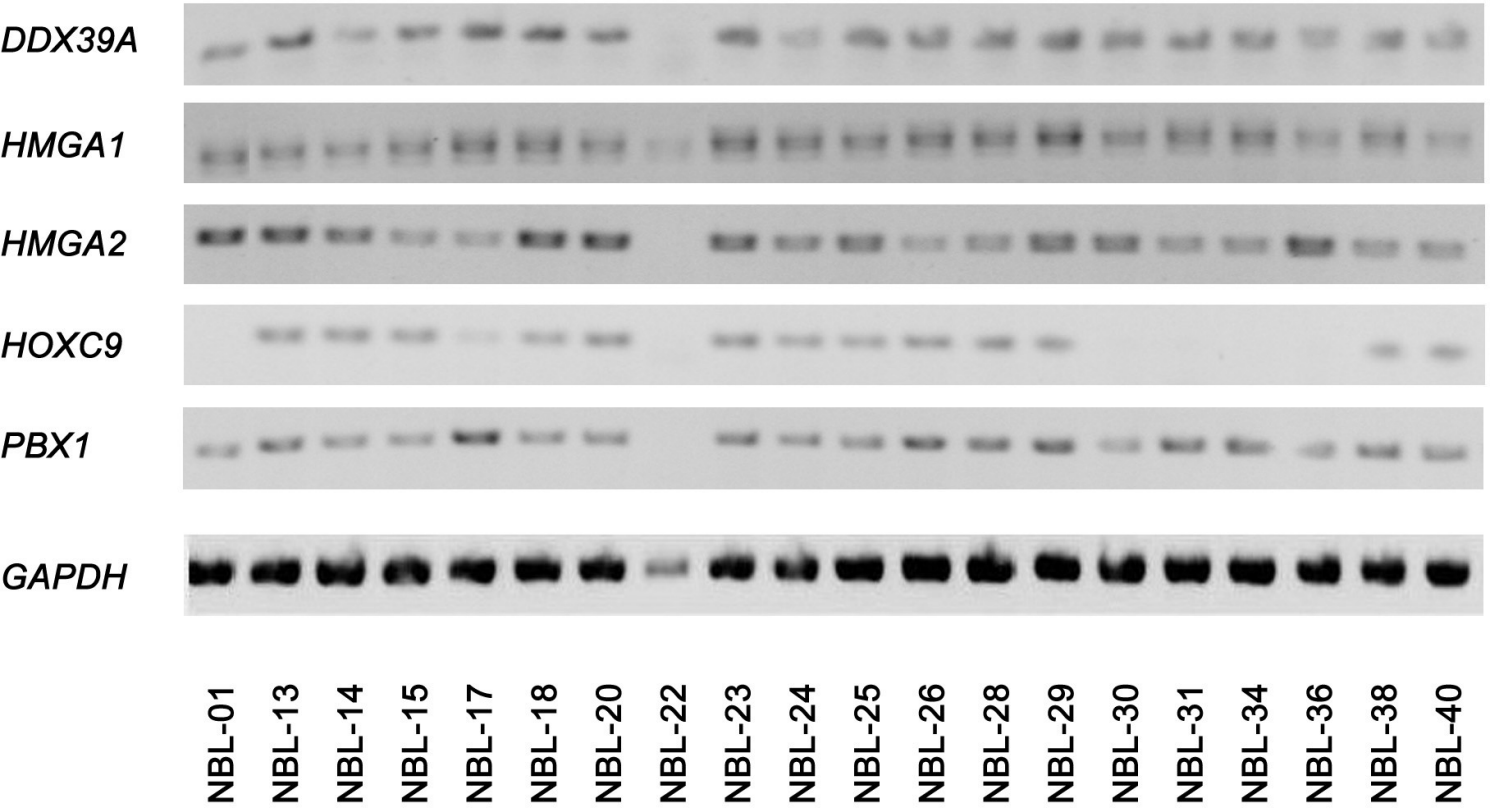
Analysis of endogenous gene expressions of putative biomarkers in patient-derived NBL cell lines. Representative gel electrophoresis images captured using a UV transilluminator are shown for each sample, including *GAPDH* as a reference housekeeping gene.

**Fig 3:**
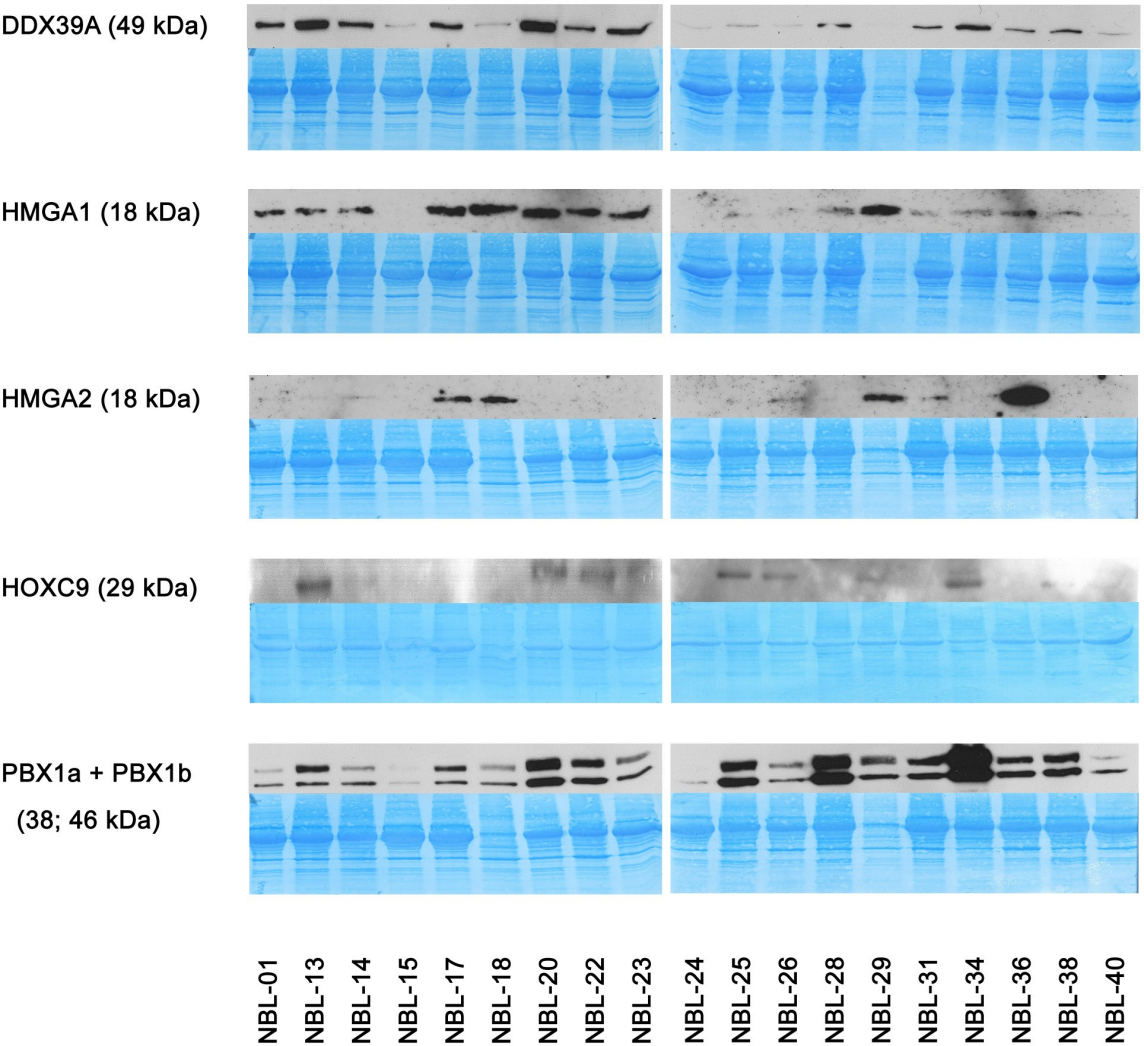
Analysis of endogenous protein levels of putative biomarkers in patient-derived NBL cell lines. Representative immunoblot images captured using X-ray film are shown for each sample. Since looking for reliable loading control protein that would provide satisfactory results throughout all analyzed NBL cell lines was showed to be difficult, loaded amounts of proteins for each sample are documented on the corresponding PVDF membrane scan. As the NBL-30 cell lysate was not obtained in the appropriate quality, this cell line was finally excluded from this screening.

A cohort consisting of 20 patients suffering from NBL was included in this study: a detailed clinical description of these patients is given in the Table 3. In this cohort, we analyzed the expression of putative biomarkers in a set of FFPE tumor samples using IHC and in corresponding cell lines (i.e. cell lines derived from these tumor samples) using WB and RT-PCR. Complete detailed results of this screening in FFPE samples are given in the Table 4 and representative micrographs together with positive controls are also showed (Fig 1). Expression of these biomarkers at mRNA (Fig 2) and protein (Fig 3) levels in corresponding cell lines were also analyzed in this part of the study. In general, high levels of DDX39A and PBX1 were found in all tumor samples while HMGA proteins showed poor (HMGA1) or no (HMGA2) endogenous expression in tumor cells (Table 4). HOXC9 protein was detected at various levels in different tumor samples regardless any clinical parameter listed in the Table 3. In contrast, expression patterns of these putative biomarkers on the protein level varied through the panel of NBL cell lines (Fig 3). Interestingly, although HMGA2 protein was not detectable by IHC in any tumor sample, we observed *HMGA2* mRNA levels in most of the cell lines (Fig 2) and apparently high levels of this protein in several of them (Fig 3).

**Table 3:** Clinical description of the patients included in this study. Age at the time of biopsy (in months). Tumor histology according Shimada system: UH, unfavorable histology; FH, favorable histology; N/A, not available. INSS stage according to the International Neuroblastoma Staging System Committee (INSS) system. NBL risk category: LR, low risk; IR, intermediate risk; HR, high risk. Status: NED, no evidence of disease; DOD, dead of disease; AWD, alive with disease.

Sample No.	Age (months)	Tumor histology	INSS stage	NBL risk category	Status	Corresponding cell line
1	1	FH	2A	LR	NED	NBL-01
2	13	FH	2A	LR	NED	NBL-13
3	6	FH	2B	LR	NED	NBL-14
4	5	UH	3	IR	NED	NBL-15
5	102	FH	2A	LR	NED	NBL-17
6	236	UH	4	HR	DOD	NBL-18
7	37	UH	4	HR	AWD	NBL-20
8	11	UH	3	IR	NED	NBL-22
9	6	UH	4	IR	NED	NBL-23
10	42	N/A	4	HR	DOD	NBL-24
11	1	FH	1	LR	NED	NBL-25
12	6	FH	1	LR	NED	NBL-26
13	1	FH	2B	LR	NED	NBL-28
14	60	UH	4	HR	DOD	NBL-29
15	3	FH	4	IR	NED	NBL-30
16	26	UH	1	LR	NED	NBL-31
17	16	UH	4	HR	AWD	NBL-34
18	10	UH	3	HR	NED	NBL-36
19	31	UH	4	HR	NED	NBL-38
20	36	UH	4	HR	NED	NBL-40

**Table 4:** Results of IHC analyses of DDX39A, HMGA1, HMGA2, HOXC9, and PBX1 expression in tumor samples. The percentage of antigen-positive tumor cells (TC) was counted and categorized into five levels: - (0% positive TC), +/- (1-10% positive TC), + (11-50% positive TC), ++ (51–80% positive TC), and +++ (81–100% positive TC). The intensity of immunostaining (immunoreactivity, IR) was classified as none (0), weak (1), medium (2), or strong (3). PQ, poor quality of the specimen (impossible to perform a correct evaluation for the respective biomarker). Sample No. 13 was not available for this IHC analysis, sample No. 14 was excluded due to its overall poor quality.

Sample No.	DDX39A		HMGA1		HMGA2		HOXC9		PBX1	
	% TC	IR	% TC	IR	% TC	IR	% TC	IR	% TC	IR
1	+++	3	-	0	-	0	+++	1	+++	2
2	+++	3	+	1	-	0	+++	1	+++	3
3	+++	3	+	1	-	0	+++	1	+++	3
4	+++	3	-	0	-	0	+	0-1	+++	2
5	++	2	-	0	-	0	++	2	++	2
6	+++	2	-	0	-	0	+++	1-2	+++	3
7	+++	3	+/-	2	-	0	+	1	+/-	1
8	PQ		-	0	-	0	+/-	1	PQ	
9	+++	3	-	0	-	0	+	1	++	3
10	++	2	-	0	-	0	+	1	+++	3
11	+++	3	-	0	-	0	+	2	+++	2
12	+++	3	-	0	-	0	++	2	+++	2
15	+++	3	-	0	-	0	++	2	+++	3
16	+++	3	-	0	-	0	+	1	+++	3
17	PQ		-	0	-	0	PQ		+++	3
18	+++	3	+	1	-	0	++	1	+++	3
19	PQ		++	2	-	0	PQ		PQ	
20	++	3	+++	2	-	0	-	0	+++	2

### Screening of resistance to retinoids was used for selection of subset of cell lines for experiments

All 20 NBL cell lines included in this study were categorized as more and less sensitive to retinoids according to the results of evaluation of their proliferation activity after treatment with natural and synthetic retinoids. Because IC_50_ calculation was not proven effective in our experimental setting, we evaluated the non/responsiveness to retinoids based on proliferation activity as measured by MTT assay (Table 5). For the next part of this study, six selected NBL cell lines were sorted into retinoid-sensitive (NBL-13, NBL-28, NBL-40) and retinoid resistant (NBL-17, NBL-25, NBL-36) groups according to the ability of retinoid to inhibit cell proliferation.

**Table 5:** Results of MTT assay. Cell viability was measured after the treatment with the respective retinoid compared to untreated cells. Green/red colors indicate more sensitive/resistant cell lines that were selected for the next part of this study. PQ - poor quality, * - statistical significance P < 0.05, ** - statistical significance P < 0.01. NBL-15 cell line was finally excluded from the evaluation due to overall reduced proliferation activity.

Treatment/ Cell line	Control	ATRA	9-*cis*-RA	13-*cis*-RA	BEX	4-HPR
NBL-01	1	0.95	0.82**	0.85**	0.99	0.84*
NBL-13	1	0.65**	0.48**	0.54**	0.48**	0.53**
NBL-14	1	0.75**	0.61**	0.59**	0.56**	0.52**
NBL-17	1	0.87	0.83	0.85	0.98	1
NBL-18	1	0.54**	0.46**	0.62**	0.32**	1.07
NBL-20	1	1.01	0.88	0.95	1.13	0.98
NBL-22	1	0.65*	0.57**	0.58**	0.74*	0.71*
NBL-23	1	1	0.69	0.9	0.51*	0.83
NBL-24	1	0.87	0.73**	0.81*	0.81*	0.84*
NBL-25	1	1.08	1.04	1.06	1.1	0.73**
NBL-26	1	0.86	0.71*	0.77	0.81	0.75*
NBL-28	1	0.61**	0.51**	0.59**	0.66**	0.53**
NBL-29	1	0.91*	0.86*	0.79**	0.79**	0.95
NBL-30	1	0.97	0.89	0.92	0.85	1.03
NBL-31	1	1.09	0.96	1.12	1.01	0.78**
NBL-34	1	0.96	0.76**	0.91	0.86	0.84*
NBL-36	1	0.78**	0.8**	0.84**	0.82**	0.89*
NBL-38	1	1.06	0.69	0.95	0.71	0.75
NBL-40	1	0.64**	0.63**	0.7**	0.85**	0.74**

### Putative biomarkers of retinoid response in sensitive and resistant NBL cell lines show different expression patterns after treatment with natural and synthetic retinoids

In the next part of this study, the subset of six patient-derived cell lines as described above and two reference cell lines were treated with natural or synthetic retinoids. Natural retinoids included ATRA, 9-*cis*-RA, and 13-*cis*-RA. Bexarotene and Fenretinide were chosen as examples of synthetic retinoids.

Extensive analysis of the expression of all candidate biomarkers in all cell lines on both the mRNA and protein level revealed that retinoids are able to significantly affect the expression of the examined putative biomarkers, especially at the protein level. Moreover, our results suggest that the response to experimental treatment may vary depending on the sensitivity/resistance of a particular cell line to retinoids and on the retinoids used in the experiment (natural vs. synthetic). An overview of all results obtained in this study is given in Table 6.

**Table 6:** An overview of detected changes in putative marker expression in sensitive and resistant NBL cell lines after treatment with natural or synthetic retinoids. The upward arrow indicates an increase in expression of the respective mRNA and/or protein, and the downward arrow indicates a decrease in mRNA and/or protein expression.

Putative marker	mRNA		Protein	
	Natural retinoids	Synthetic retinoids	Natural retinoids	Synthetic retinoids
**DDX39A**	Not changed	Not changed	**↑In sensitive patient-derived** **cell lines**	**↑In sensitive** **patient-derived** **cell lines**
			Not changed in resistant and reference (sensitive) cell lines	Not changed in resistant and reference (sensitive) cell lines
**HMGA1**	Various response	Various response	Various response in sensitive patient-derived cell lines	**↑In all cell lines**
**HMGA2**	**↓In all cell lines**	Various response	Poor expression	Poor expression
**HOXC9**	Not changed	Not changed	**↑In all** **patient-derived cell lines**	Various response
			Not changed in reference cell lines	
**PBX1**	**↑In sensitive cell lines**	Not changed in sensitive cell lines	**↓In sensitive** **patient-derived cell lines**	**↓In sensitive** **patient-derived cell lines**
	Various response in resistant cell lines	**↓ In resistant cell lines**	Various response in reference and resistant cell lines	Various response in reference and resistant cell lines

In general, RA-sensitive cell lines (NBL-13, NBL-28, NBL-40, SH-SY5Y, SK-N-BE(2)) responded to the treatment by upregulating the expression of the respective mRNA or protein, whereas downregulation of mRNA levels was observed in RA-resistant cell lines (NBL-17, NBL-25, NBL-36). No change in expression or various responses to particular retinoids was detected for some of the analyzed biomarkers.

### Treatment with retinoids results in consistent changes in *HMGA2* and *PBX1* gene expression

**Fig 4:**
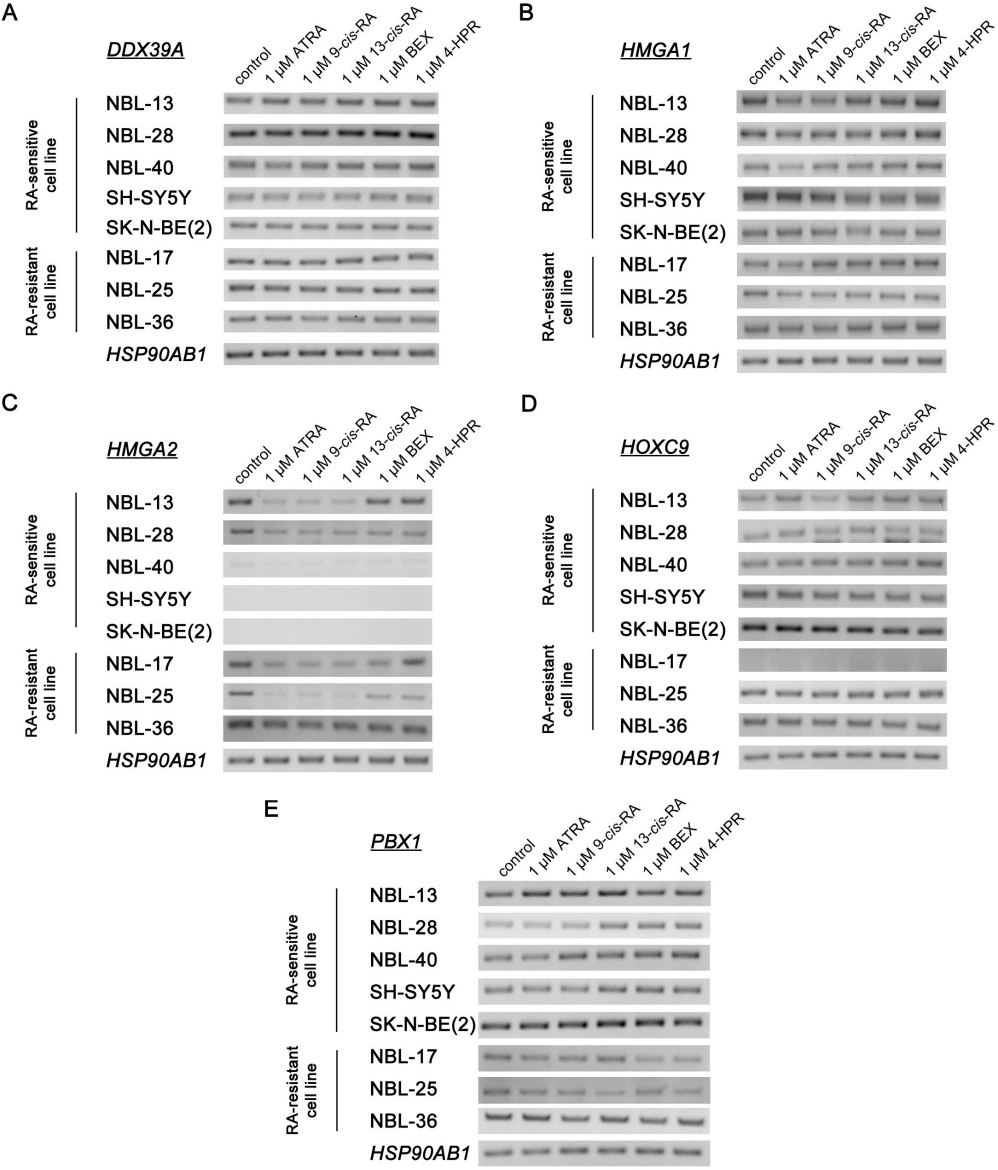
Analysis of candidate biomarkers mRNA expression. *DDX39A* (A), *HMGA1* (B), *HMGA2* (C), *HOXC9* (D), and *PBX1* (E). Relative gene expression was determined in 6 patient-derived and 2 reference cell lines after 7 days of treatment with natural (ATRA, 9-*cis*-RA, 13-*cis*-RA) and synthetic (BEX, 4 HPR) retinoids administered at a 1 μM concentration. Experiments were repeated in triplicate. Representative gel electrophoresis images captured using a UV transilluminator are shown for each sample, including *HSP90AB1* as a reference gene.

The first part of our study focused on retinoid-mediated changes in the expression of candidate biomarkers on the mRNA level after exposure to 1 of 5 types of retinoids (ATRA, 9-*cis*-RA, 13-*cis*-RA, BEX, 4-HPR) at the same concentration of 1 μM for 7 days (Fig 4).

Among the examined biomarkers, the biggest effect of retinoids on mRNA expression was identified in the case of *HMGA2*. In all cell lines where endogenous expression of *HMGA2* was detected (NBL-13, NBL-28, NBL-17, NBL-25, NBL-36), significant downregulation of this gene after treatment with natural retinoids was observed. In contrast, treatment with synthetic retinoids did not show a consistent effect on *HMGA2* expression among the analyzed cell lines, and both up- and downregulation were observed (Fig 4C).

All NBL cell lines included in this study expressed *PBX1* mRNA. In RA-sensitive cell lines, treatment with natural retinoids resulted in *PBX1* upregulation, while RA-resistant cell lines responded to the same treatment mainly by *PBX1* downregulation, especially when synthetic retinoids were used (Fig 4E). Graphs showing the relative gene expressions of examined biomarkers with indicated significant changes are presented in the Supporting information (S2 Fig).

### DDX39A1 and PBX1 proteins show consistent changes in expression after retinoid treatment among RA-sensitive NBL cell lines

**Fig 5:**
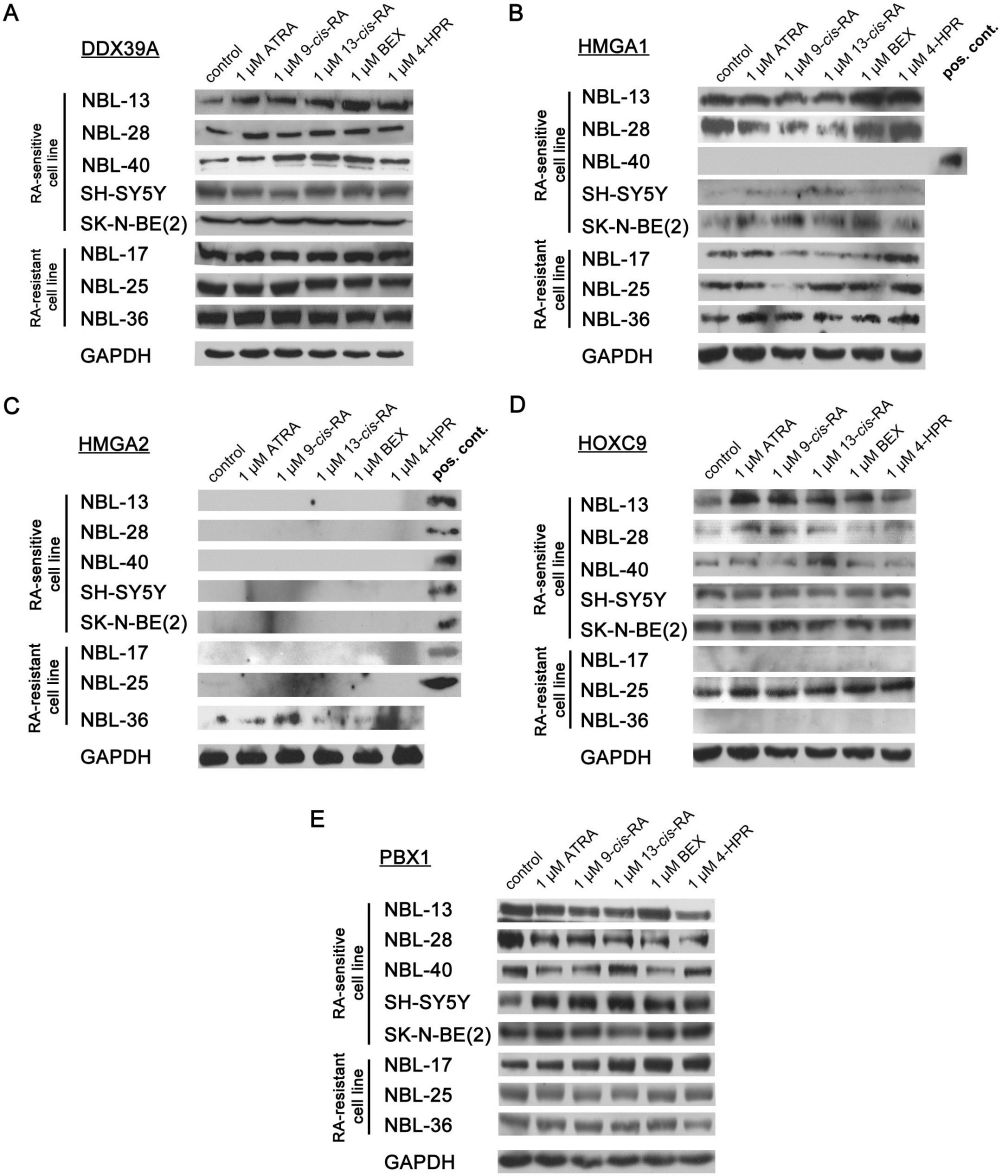
Analysis of candidate biomarkers protein expression. (A) DDX39A–49 kDa; (B) HMGA1–18 kDa; (C) HMGA2–18 kDa; (D) HOXC9–29 kDa; and (E) PBX1–46 kDa. Relative protein expression was determined in 6 patient-derived and 2 established NBL cell lines after treatment with natural (ATRA, 9-*cis*-RA, 13-*cis*-RA) and synthetic (BEX, 4-HPR) retinoids administered at a 1 μM concentration for 7 days. Images of the representative immunoblot captured using X-ray film are shown for each sample, including GAPDH as a reference protein. Experiments were repeated in triplicate.

We subsequently performed Western blot analysis of candidate biomarkers after treatment with retinoids for 7 days. Our analysis revealed that retinoid-mediated changes in biomarker expression occurred more frequently at the protein level than at the mRNA level and that some of the analyzed proteins show specific expression patterns in only RA-sensitive subset of cell lines. Among all examined cell lines, upregulation of protein expression compared to that in control cells was the most frequent response to retinoid treatment (Fig 5). Graphs showing the relative protein levels of examined biomarkers with indicated significant changes are presented in the Supporting information (S3 Fig).

We detected DDX39A overexpression in RA-sensitive patient-derived cell lines after both natural and synthetic retinoids (Fig 5A). Interestingly, we did not observe retinoid mediated upregulation of DDX39A protein in SH-SY5Y and SK-N-BE(2) reference cell lines that are also sensitive to retinoids. In NBL-25 and NBL-36 RA-resistant cell lines, DDX39A was slightly downregulated after treatment with synthetic retinoids.

HMGA1 protein was detected in all cell lines except for the NBL-40 RA-sensitive cell line (Fig 5B). On one hand, upregulation was detected in all cell lines expressing HMGA1 when synthetic retinoids were used. On the other hand, treatment with natural retinoids caused various responses in the examined cell lines. Both up- and downregulation of HMGA1 were observed, and therefore, no specific expression pattern indicating sensitivity or resistance to retinoids was identified in this case.

Analysis of HMGA2 protein expression revealed that only the NBL-36 RA-resistant cell line was HMGA2-positive (Fig 5C). Even in this cell line, HMGA2 protein expression was relatively poor, and we were therefore unable to properly evaluate its response to retinoid treatment. Nevertheless, HMGA2 positivity seems to indicate the RA-resistant phenotype in NBL cell lines.

We further analyzed the RA-mediated change in HOXC9 expression that was detected in all cell lines except NBL-17 and NBL-36 RA-resistant cell lines (Fig 5D). Treatment with natural retinoids resulted in HOXC9 upregulation in all patient-derived cell lines with detectable HOXC9 expression, and this pattern was more notable in RA-sensitive cell lines.

PBX1 protein expression was downregulated in RA-sensitive patient-derived cell lines treated with both natural and synthetic retinoids. In RA-resistant cell lines, namely, in the NBL-17 cell line, PBX1 was slightly upregulated, especially after treatment with synthetic retinoids (Fig 5E).

## Discussion

Our study aimed to analyze the expression of five candidate biomarkers of sensitivity/resistance to retinoids (DDX39A, HMGA1, HMGA2, HOXC9, PBX1) in a single experimental study using a set of patient-derived and reference NBL cell lines. Our results suggest that on the mRNA level, 7 days of exposure to natural and synthetic retinoids resulted in a change in expression of 3 out of 5 analyzed genes (*HMGA1*, *HMGA2*, and *PBX1*), which could indicate ongoing changes towards a more differentiated phenotype mediated by retinoids. Further analysis of protein expression by Western blotting revealed that retinoids modulated the expression of the studied biomarkers at the protein level in an even more significant manner.

DDX39A, a protein that was very recently reported to be highly expressed in undifferentiated cells and NBL patients with poor prognosis, was analyzed in this study with the aim of determining its role in predicting the response to retinoids [12,20]. While no difference in the expression of DDX39A mRNA was observed in treated cells compared to control cells, Western blot analysis showed that both natural and synthetic retinoids were able to upregulate the expression of DDX39A protein in the RA-sensitive subset of NBL cell lines derived in our laboratory (NBL-13, NBL-28, NBL-40). The obtained results were therefore in conflict with previously published research [12] that analyzed DDX39A protein levels in 2 reference NBL cell lines and primary tumor samples before and after treatment with 10 μM ATRA and observed higher DDX39A expression in undifferentiated cells. Since several experiments in our project suggested that reference and patient derived cell lines with comparable sensitivity to retinoids may show different expression patterns for the examined biomarkers, this discrepancy could be explained at least partially by the difference in the type of experimental material. Therefore, more extensive analysis with larger groups of cell lines and tumor samples from patients treated with retinoids is needed to validate the possible role of DDX39A protein as a biomarker of response to retinoids besides its indicated prognostic value.

We further evaluated the expression of two candidate biomarkers from the high mobility group A protein family: HMGA1 and HMGA2. The HMGA gene family was suggested to be a new family of oncogenes important in the pathogenesis of human cancer, since their increased expression was found to correlate with neoplastic transformation in several human cancers. In particular, *HMGA2* was already shown to be involved in determining RA resistance in NBL cell lines in 1999 [13]. In the present study, *HMGA1* mRNA was expressed in all examined cell lines, which is in accordance with previously published results [20]. Among the 5 RA-sensitive cell lines examined in our study, 3 were patient derived, which we believed could help better validate the usefulness of the selected candidate biomarkers in the prediction of response to retinoids. According to the results we obtained, no clear *HMGA1* expression pattern was shown to be related to the responsiveness to retinoids neither in RA-sensitive nor RA-resistant subsets of cell lines. Moreover, the change in HMGA1 expression after treatment with retinoids was relatively low compared to that in untreated cells. We therefore cannot confirm a previously published study that observed ATRA-mediated *HMGA1* downregulation in RA-sensitive NBL cell lines and *HMGA1* upregulation in cell lines resistant to ATRA [13]. However, it should be noted that in the study mentioned above, reference NBL cell lines were treated with ATRA for 48 h, whereas in our experiments, cells were exposed to retinoids for 7 days. Therefore, retinoid-mediated changes in *HMGA1* expression seem to be more notable during the first few days following retinoid administration.

Apart from HMGA1 analysis, we also examined the expression of a second member of the HMGA family – HMGA2. Previously, a study reported that *HMGA2* mRNA expression was detected only in NBL cell lines resistant to retinoids, suggesting its potential function in predicting the response to retinoids [13]. All 3 RA-resistant cell lines used in our study express *HMGA2*. However, our detailed analysis also revealed detectable levels of *HMGA2* mRNA in 2 RA-sensitive cell lines (NBL 13, NBL 28). We consequently concluded that *HMGA2* positivity is insufficient in predicting resistance to retinoids *in vitro*; however, all *HMGA2* negative cell lines showed sensitivity to retinoids. Therefore, our data suggest that the absence, rather than presence, of *HMGA2* expression could predict the cellular response to retinoids. Furthermore, this study revealed that natural but not synthetic retinoids caused significant downregulation of the *HMGA2* gene in all cell lines where *HMGA2* was detected. We subsequently performed HMGA2 expression analysis at the protein level, which showed that this protein was completely missing in the NBL cell lines included in this study except for the NBL-36 RA-resistant cell line. Using the HMGA2-positive phenotype as a marker of resistance to retinoids would therefore produce false-negative results, as remaining-RA resistant, HMGA2-negative cell lines would be identified as RA-sensitive. Further study with more cell lines and tumor samples could help to better judge HMGA2 involvement in maintaining resistance to retinoids besides its apparent negative prognostic value in the various tumor types mentioned above.

The transcription factor HOXC9 is discussed as a regulator of differentiation course within a cell [21] and was also found to induce G1 arrest in NBL cells. Moreover, the *HOXC9* promoter was epigenetically primed in an active state in ATRA-sensitive reference NBL cell lines and silenced in ATRA-resistant cell lines according to another study, indicating its possible use as a marker of sensitivity to retinoids [14]. In the same study, HOXC9 protein levels were elevated in differentiated cells compared to those undergoing ATRA-induced differentiation. The role of HOXC9 in indicating better patient outcome was also supported by other studies on glioblastoma [22] and breast carcinoma [23]. In our study, *HOXC9* mRNA was detected in all RA-sensitive and 2 out of 3 RA-resistant cell lines, indicating that *HOXC9* promoter silencing did not generally occur in all cell lines with an RA-resistant phenotype, as suggested in the previously mentioned study. Because we used patient-derived cell lines, whereas reference cell lines were used in the study by Mao and coworkers, a more detailed analysis of the *HOXC9* promoter in larger groups of NBL cell lines could help confirm these results. Western blot analysis of HOXC9 protein expression also showed promising results, supporting previous reports on the key role of HOXC9 in NBL differentiation [14,24]. In 2 of 3 RA-resistant cell lines, HOXC9 was not detected, and in RA-sensitive cell lines, HOXC9 was upregulated more significantly in cells treated with retinoids than in those showing RA resistance. Based on these results together with studies mentioned above, HOXC9 negative cell lines could be less prone to retinoid-mediated differentiation, and the HOXC9 negative phenotype could therefore indicate resistance to retinoids. However, the opposite trend was not observed – not all HOXC9-positive cell lines were sensitive to retinoids, suggesting that analysis of endogenous HOXC9 expression itself is insufficient in reliable determining RA responsiveness in NBL cell lines.

PBX1, which was recently suggested to increase sensitivity to 13-*cis*-RA, was also analyzed in the present study. Previously, published results indicated its upregulation in RA-sensitive cells only, and a study reported that PBX1 downregulation resulted in a more aggressive phenotype [17]. Our experiments showed that natural retinoids caused a significant upregulation of *PBX1* mRNA in some RA-sensitive cell lines (NBL-13, SK-N-BE(2)), which is in accordance with the study mentioned above. However, we did not observe the same trend in the 3 remaining RA-sensitive cell lines. Compared to research we just mentioned, in which 10 μM 13-*cis*-RA was administered, all retinoids involved in our project were used at a concentration of 1 μM, which could also have influenced the results. Subsequent analysis of PBX1 expression at the protein level revealed a slight downregulation of this protein in RA-sensitive cell lines. This part of our experiment is different from the study mentioned above, since we did not also confirm the RA-mediated upregulation of PBX1 in RA-sensitive cell lines at the protein level, but PBX1 protein expression is likely downregulated. In addition to NBL, PBX1 was recently extensively studied in other types of cancer, and some of those studies suggested the opposite function of PBX1 in cancer progression that could be in accordance with the results we obtained [25,26].

Our study aimed to perform an extensive analysis of several candidate biomarkers of RA responsiveness and confirm the potential use of some of these gene products and their corresponding proteins as indicators of resistance or sensitivity to retinoids. Analysis of the expression of these markers in combination might be more useful than analysis of these markers individually. Among all examined biomarkers, endogenous HMGA2 positivity together with HOXC9 negativity could predict a poor response to retinoid treatment. Moreover, cells undergoing RA-mediated differentiation could be identified by upregulation of *PBX1* mRNA and/or DDX39A and HOXC9 proteins.

## Conclusions

In summary, our study was the first to evaluate the predictive value of five selected protein biomarkers in the same set of NBL cell lines after treatment with natural or synthetic retinoids. We found that cell lines that were HMGA2-positive and/or HOXC9-negative have a reduced sensitivity to retinoids. Furthermore, our experiments revealed that the retinoid-sensitive cell lines showed a uniform pattern of change after treatment with both natural and sensitive retinoids: increased DDX39A and decreased PBX1 protein levels. Our results proved that in NBL cells, these putative protein biomarkers are clearly associated with retinoid sensitivity or resistance, and the endogenous or induced expression of these markers can distinguish between the two phenotypes.

## Supporting information

S1 Fig Examples of initial RT-PCR saturation analysis. For each gene of interest, two different samples, i.e. two different cell lines were chosen to optimize the number of cycles used for semi-quantitative gene expression analysis. Cell lines used in this analysis: NBL-13 (Sample 1 for *HMGA1*, Sample 2 for *DDX39A*, *HMGA2*, *PBX1*), NBL-36 (Sample 1 for *HMGA2*, Sample 2 for *HMGA1*), NBL-40 (Sample 1 for *DDX39A*), SK-N-BE(2) (Sample 1 for *HOXC9*, *PBX1*), SH-SY5Y (Sample 2 for *HOXC9*).
(EPS)

S2 Fig **Relative band density for *DDX39A* (A), *HMGA1* (B), *HMGA2* (C), *HOXC9* (D), and *PBX1* (E) mRNA expression.** The data are presented as the mean + SD, experiments were repeated in triplicate. The black line crossing the 100% represents the relative band density in untreated cells. * p< 0.05 indicates a significant difference compared to untreated cells. RA sensitive cell lines are illustrated in green and RA-resistant cell lines in red color. ImageJ software was used for visualized bands quantification.
(EPS)

S3 Fig **Relative DDX39A (A), HMGA1 (B), HMGA2 (C), HOXC9 (D), and PBX1 (E) protein expression.** The data are presented as the mean + SD, experiments were repeated in triplicate. The black line crossing the 100% represents the relative biomarker expression in untreated cells. * p< 0.05 indicates a significant difference compared to untreated cells. RA sensitive cell lines are illustrated in green and RA-resistant cell lines in red color. ImageJ software was used for visualized bands quantification.
(EPS)
